# Characterization of Cellulase Secretion and Cre1-Mediated Carbon Source Repression in the Potential Lignocellulose-Degrading Strain *Trichoderma asperellum* T-1

**DOI:** 10.1371/journal.pone.0119237

**Published:** 2015-03-05

**Authors:** Qun Wang, Hui Lin, Qi Shen, Xiaoping Fan, Naling Bai, Yuhua Zhao

**Affiliations:** 1 Institute of Microbiology, College of Life Sciences, Zhejiang University, Hangzhou, China; 2 Institute of Environment Resource and Soil Fertilizer, Zhejiang Academy of Agriculture Science, Hangzhou, China; University of Wisconsin-Milwaukee, UNITED STATES

## Abstract

*Trichoderma asperellum*, a traditional bio-control species, was demonstrated to be an excellent candidate for lignocellulose degradation in this work. Comparing to the representatively industrial strain of *Trichoderma reesei*QM6a, *T. asperellum* T-1 showed more robust growth, stronger spore production, faster secretion of lignocellulose-decomposing enzymes and better pH tolerance. The reducing sugar released by strain T-1 on the second day of fermentation was 87% higher than that of strain QM6a, although the maximum reducing sugar yield and the cellulase production persistence of the strain T-1 were lower. Our experiment found that the cellulase secretion was strongly inhibited by glucose, suggesting the existence of carbon source repression pathway in *T. asperellum* T-1. The inhibiting effect was enhanced with an increase in glucose concentration and was closely related to mycelium growth. SDS-PAGE and secondary mass-spectrum identification confirmed that the expression of endo-1,4-β-xylanase I in *T. asperellum* T-1 was down-regulated when glucose was added. The factor Cre1, which plays an important role in the down-regulation of the endo-1,4-β-xylanase I gene, was investigated by bioinformatics methods. The protein structure of Cre1, analyzed using multiple protein sequence alignment, indicates the existence of the Zn-fingers domain. Then, the binding sites of Cre1 on the endo-1,4-β-xylanase I gene promoter were further elucidated. This study is the first report about Cre1-mediated carbon repression in the bio-control strain *T. asperellum* T-1. All of the above results provided good references for better understanding *T. asperellum* T-1 and improving its application for lignocellulose degradation.

## Introduction

Biomass energy, the fourth-largest energy source beyond coal, oil, and natural gas, occupies an important role in the global energy system. The most abundant biomass resources existing in nature are the agricultural wastes and the sideline products rich in cellulose, hemicellulose and lignin. These wastes and products can be converted into acids, hydrocarbons and their derivatives through microbial fermentation. Currently, the tremendous commercial potential of cellulases in a variety of applications, especially in microbial energy development, remains the driving force in the research area of lignocellulose utilization [1]. More important is that a constant increase in energy consumption, the depletion of fossil fuels, and increasing environmental concerns have globally shifted the focus to generate energy from non-renewable carbon sources [2]. As an agricultural country, China has enormously renewable lignocellulose resources, with a yield of (6–8)*10^8^ tons per year [3]. The degradation and utilization of lignocellulose can not only solve the environmental problem, but also provide substantial social benefits and ease the energy crisis.

The main problem in lignocellulose utilization is the contradiction between the high demand for using lignocellulosic biomass as a fermentation substrate and the high cost of converting it into fermentable sugars [4]. Microbial conversion with the advantages of no pollution and low cost has attracted tremendous attention. Filamentous fungi have shown outstanding advantages in lignocellulose utilization because of their capacity to secrete large amounts of lignocellulosic enzymes, especially *Trichoderma* spp.[5–8]. They can release fermentable sugars from plant cell walls [9], and this ability has been exploited in industry to produce cellulases in quantities exceeding 100 g L^-1^ of culture [10].

*Trichoderma asperellum*, frequently isolated from soil, plant roots and tissues, fungal biomass, and wood, is an effective biological control agent against many pathogenic microorganisms, such as *Rhizoctonia solani*, *Fusarium sporotrichioides* and *Fusarium oxysporum* [11, 12]. In recent years, *T. asperellum* has received considerable attention as a model bio-control species to explore the mechanism of reducing plant disease. However, its application potential in lignocellulose degradation has not been thoroughly investigated. In fact, for most plant-pathogenic fungi, breaking down plant cell walls is of major importance to invade host tissues and to obtain nutrients [13]. Because the plants, such as wheat straw in our research, are mostly composed of cellulose and hemicelluloses, the cellulolytic activities of the bio-controlling *Trichoderma* is inseparable for their competition with plant pathogens. This theory was also evaluated as a possible mechanism for obtaining energy resources [14]. Currently, even though there are many filamentous fungi with strong abilities to degrade lignocellulose, different strains have their own advantages. For example, *T. reesei* has the strongest total enzyme activity, although it has a low number and a poor diversity of genes encoding enzymes likely to be involved in biomass degradation; *Phanerochaete chrysosporium* has a superior ability to degrade lignin, as it can secrete a wide range of lignin peroxidases and glycosyl hydrolases (GHs). In addition, *Aspergillus niger* produces more types of cellulases compared with other filamentous fungi [15]. Therefore, enzyme cocktails and co-fermentation become effective approaches to make full use of the advantages of different fungi. On the other hand, exploring new cellulose-degrading fungi is necessary and may bring new valuable applications.

In our previous research [16], a strain called *Trichoderma* sp. T-1, which is abundant in cellulases, showed a strong ability to convert wheat straw into fermentable sugars, and it was used as the main fermentation strain (92%) in a fungal consortium to saccharify wheat straw. After that, *Trichoderma* sp. T-1 was identified as *T. asperellum* and was continually used in the further study of lignocellulose degradation. Actually, *T.asperellum* has already been used to degrade lignocellulose, especially in the region where this filamentous fungus is widespread. In Malaysia, *T. asperellum* UPM1 and *Aspergillus fumigatus* UPM2 isolated from rotten oil palm fruit bunches were used in the enzymatic hydrolysis of oil palm empty fruit bunches (OPEFB), an example of a local lignocellulose resource, with a yield of fermentable sugars up to 0.17 g g^-1^. Then, the obtained sugars were further used as carbon sources in ethanol production by yeast [17].

Here, we performed a specific study on the lignocellulose degradation ability of *T. asperellum* T-1 by comparing with the industrial standard strain *T. reesei* QM6a to highlight the advantages of T-1 in lignocellulose degradation. In addition, the first systematic and comprehensive study on carbon source repression in *T. asperellum* T-1 was carried out to preliminarily explore the regulation of cellulase expression in *T. asperellum* T-1. All of these efforts provided guidance for the further and better application of *T. asperellum* T-1 in lignocellulose degradation.

## Materials and Methods

### Strains and pretreatment of wheat straw

*T. asperellum* T-1 (GenBank: KM277355) was isolated from African jungle soil samples collected from the Republic of Cameroon and preserved in the Lab of Microbiology, Zhejiang University. *T. reesei* QM6a (DSM No.: 768) was bought from DSMZ (German Collection of Microorganisms and Cell cultures). Both of the strains were activated on potato-dextrose agar (PDA).

Wheat straw, collected from Bozhou city, Anhui province (GPS coordinates: 33.923136, 115.83361) was dried before milling through sifters to ensure that the particle diameter was between 75 μm and 154 μm.

### Ethics statements

Wheat straw, collected from Bozhou city, Anhui province (GPS coordinates: 33.923136, 115.83361) was agricultural waste, and no specific permissions were required for the sampling activity. The field studies did not involve endangered or protected species.

### Culture and enzyme production of *T. asperellum* T-1 and *T. reesei* QM6a

Both *T. asperellum* T-1 and *T. reesei* QM6a were inoculated on PDA plates for 5–7 days. After that, spores were harvested into 0.1% (v/v) Tween 20. Obtained spores were washed with 0.1% (v/v) Tween 20 once before being suspended with sterile water. Spores of *T. asperellum* T-1 and *T. reesei* QM6a at final concentrations of 10^6^ spores ml^-1^ were inoculated into 100 ml of basal medium [in concentrations of g l^-1^: KH_2_PO_4_, 2.0; (NH_4_)_2_SO_4_, 1.4; MgSO_4_.7H_2_O, 0.3; CaCl_2_, 0.3; FeSO_4_.7H_2_0, 0.005; MnSO_4_.H_2_O, 0.00156; ZnSO_4_.7H_2_O, 0.0014; CoCl_2_, 0.002; Urea, 0.3; pH 5.5]. 1% milled wheat straw was used as the carbon source. Then, the triangular flasks were shaken at 200 rpm and 28°C. 1.5 ml of fermentation liquor was taken every 24 hours and centrifuged at a speed of 12,000 rpm at 4°C. After centrifugation, the supernatant was kept at -20°C before being used in the enzyme activity measurements. All of the experiments were performed in triplicate.

To compare the mycelium growth, 100 μl spore suspensions of *T. asperellum* T-1 and *T. reesei* QM6a at the same concentration (10^6^ spores ml^-1^) were spread on PDA plates, and photographic records of their growth were taken during different periods.

### Glucose inhibition experiment during enzyme production

The effect of carbon source repression in fermentation system of *T. asperellum* T-1 caused by glucose was carried as follows: spores of *T. asperellum* T-1 were inoculated as above with 1% wheat straw as a carbon source. Glucose was added at 0 h, 36 h and 60 h after the start of the fermentation to different final concentrations of 0.5%, 1.0%, 1.5% and 2.0% (w/v). The group without glucose addition was used as a control. Each group was performed in triplicate.

### Enzyme activity assay and sugar determination

The supernatant obtained in the enzyme production experiments and glucose inhibition experiments were used in the enzyme activity assays. The cellulase activity was measured according to the method of Ghose [18] by determining the filter paper cellulase (FPase) activity and the carboxymethylcellulase (CMCase) activity. The release of reducing sugars was assayed using the 3,5-dinitrosalicylic acid (DNS) method [19], and the enzymatic activities were expressed as the concentration of reducing sugar released from the substrate by cellulase.

In order to determine the consumption of the added glucose by *T. asperellum* T-1 in the glucose inhibition experiment, the reducing sugar content in the supernatant of the groups with glucose addition were estimated as the glucose equivalent using the 3,5-dinitrosalicylic acid (DNS) method [19].

### SDS-PAGE and protein identification

The fermented liquid from *T. asperellum* T-1 cultures obtained in the glucose inhibition experiment, were analyzed by SDS-PAGE to verify the change of protein expression under carbon source repression. Afterwards, the bands represented obvious changes in gene expression were cut out, and secondary mass-spectrum detection was used to preliminarily identify the proteins.

### Isolation of *cre* gene and multiple protein sequence alignment

The nucleic acid sequence of the *cre1* gene in *T. reesei* (Gene ID: 1848279) was used as the probe sequence to search for target homologous information in the genomic sequence of *T. asperellum* in the JGI database (http://genome.jgi.doe.gov/pages/search-for-genes.jsf?organism=Trias1). Then, polymerase chain reaction (PCR) and DNA sequencing were carried out to prove the validity of the sequence. In addition, primers (fp-cre1:5’-CCTCGCTGACCGCCGCTCAATCAC-3’ and rp-cre1: 5’-CCATAGTAGGGTATTTTTTATATCGT-3’) were designed according to the obtained sequence. Next, bioinformatic multiple sequence alignment was performed between the obtained protein sequence and its homologous sequences in *T. reesei* (Cre1; GenBank: CAA64655.1), *A. niger* (CreA; GenBank: CAL00597.1) and *Saccharomyces cerevisiae* (Mig1p; NCBI Reference Sequence: NP_011480.1) to analyze the protein structure of the obtained sequence.

### Obtaining the endo-1,4-β-xylanase I gene and analyzing its promoter

The nucleic acid sequence of the endo-1,4-β-xylanase I gene was obtained through the same method that we used for the *cre* gene above. Specifically, its highly homologous gene sequence in *Trichoderma viride* (endo-1,4-β-xylanase I gene; GenBank: AY370020.1) was used as a probe to search for target homologous information in the genomic sequence of *T. asperellum*. Then, PCR and DNA sequencing were used to identify the sequence validity (primers: fp-xyl: 5’-TTCCTATTGTGGTTTACTGC-3’ and rp-xyl: 5’- AGCTTTGATTTCCTGTGTAC-3’). The promoter region approximately 1 kb upstream of the open reading frame (ORF) encoding endo-1,4-β-xylanase I was used in the extension analysis.

## Results and Discussion

### Cell growth and enzyme production comparison between *T. asperellum* T-1 and *T. reesei* QM6a

**Fig 1:**
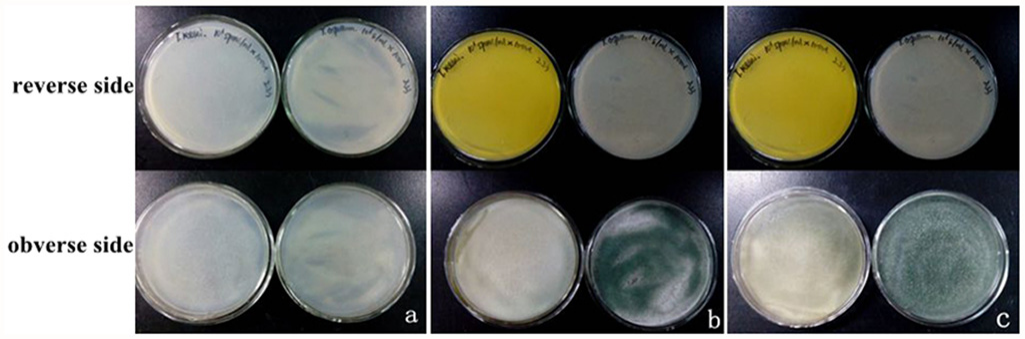
Growth comparison of *T. asperellum* T-1 (right side in a; b; c) and *T. reesei* QM6a (left side in a; b; c) on PDA both on the obverse side and the reverse side of the plates. (a) Cultivation for 1 day; (b) Cultivation for 2 days; (c) Cultivation for 5 days.

The cell growth of *T. asperellum* T-1 and *T. reesei* QM6a cultivated on PDA plates are shown in Fig. 1. *T. asperellum* T-1 exhibited a strong ability to produce spores that a large amount of spores could be observed within 2 days, while no obvious spores of *T. reesei* QM6a was detected even after 7 days of cultivation. The culture medium of *T. reesei* QM6a turned to yellow after the second day (Fig. 1b,c), but no color change was observed for *T. asperellum* T-1. One explanation for this phenomenon is that *T. reesei* may release a yellow pigment in the process of growth metabolism. Another explanation is that the overly acidic environment caused by hyphae cell metabolism may make the medium changing its color. Liquid culture experiments were conducted to verify it. Spores of *T. asperellum* T-1 and *T. reesei* QM6a with the same final concentration of 10^6^ spores ml^-1^ were inoculated into lipid potato dextrose medium and shaken at 28°C. The results showed that both *T. asperellum* T-1 and *T. reesei* QM6a had fast hyphae growth after 2 days of incubation without an obvious color change. However, green spores could be observed in bottles of *T. asperellum* T-1, which reconfirmed its stronger ability to produce spores. The pH of the fermentation liquor of *T. reesei* QM6a was approximately 4.8, while that of *T. asperellum* T-1 was 6.2. It is concluded that the color change phenomenon was caused by medium pH change during the process of cell growth. Generally, the pH change might influence the fungal enzyme production in the microbial degradation of lignocellulose. However, Sanjeev Raghuwanshi et al. found that a strain of *T. asperellum* RCK2011 showed a high cellulase production at pH values ranging from 4.0 to 10.0, indicating that the pH changes had a comparatively smaller suppression on cellulase secretion in *T. asperellum* than in other filamentous fungi. Therefore, *T. asperellum* may be more suitable for industrial production, where fermentation conditions such as pH cannot be strictly controlled to adapt to the metabolism of the microorganism.

**Fig 2:**
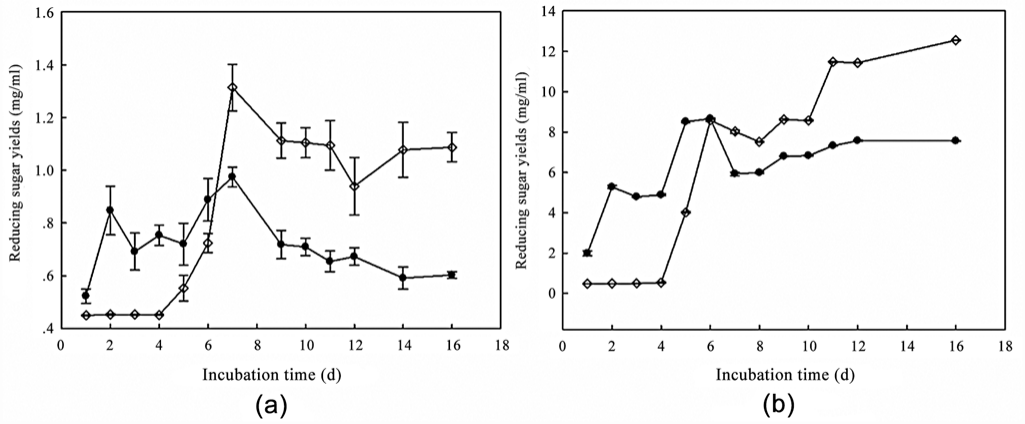
Cellulase production comparison of *T. asperellum* T-1 and *T. reesei* with wheat straw as carbon source, expressed as the concentration of reducing sugar released from the substrate by cellulase in the enzyme reaction system. (a) Filter paper activity (FPA) of *T. asperellum* T-1 (black circle) and *T. reesei* (diamond); (b) CMCase activity (endoglucanase activity) of *T. asperellum* T-1 (black circle) and *T. reesei* (diamond). Error bars denote standard deviations from the mean values of triplicate measurements (n = 3).

Furthermore, the cellulose degradation abilities of *T. asperellum* T-1 and *T. reesei* QM6a were estimated by enzyme activity assays including FPase and CMCase. FPase is a convincing marker to estimate the cellulose hydrolysis capability of fungal secretomes, and CMCase shows the activity of endoglucanase. As shown in Fig. 2, there was significant difference of the cellulose enzyme activity between *T. reesei* QM6a and *T. asperellum* T-1 and T-test analysis showed that the p value of FPA and CMCase were as high as 0.125 and 0.419, respectively. Both FPase activity and CMCase activity of *T. asperellum* T-1 exhibited a noticeable increase starting from the second day, while *T. reesei* QM6a needed four days of cultivation to show clearly rising activity in FPase and CMCase. We can speculate from Fig. 2a that the yield of reducing sugar released by strain T-1 was 87% higher than that of strain QM6a on the second day. It is possible to conclude that *T. asperellum* T-1 had a better cellulose degradation ability in the early stage of the fermentation. However, as time passed, *T. reesei* QM6a exhibited a higher cellulase enzyme activity. Additionally, the FPase activity in both *T. asperellum* T-1 and *T. reesei* QM6a decreased starting from the 7^th^ day of incubation, but *T. reesei* QM6a showed a relatively better performance. Similarly to FPase, the activity of the endoglucanase (CMCase) declined starting from the 6^th^ day in *T. asperellum* T-1, while it kept increasing in *T. reesei* QM6a. It is easy to be concluded that *T. asperellum* T-1 showed a much faster secretion of lignocellulose-decomposing enzymes but a poorer reducing-sugar yield and enzyme production persistence. And this conclusion was in agreement with the solid-state fermentation results in Sanjeev Raghuwanshi’s study [2]. They found that even with a comparatively longer fermentation period, *T. reesei* still showed superior cellulose degradation with its stronger enzyme activity and better cellulase production persistence compared with *T. asperellum* RCK2011.

In general, *T. asperellum* T-1 showed robust growth, good spore production ability, rapid cellulase secreting, and better pH tolerance during lignocellulose degradation. However, the total cellulase production yield and the production persistence of *T. asperellum* T-1 still need to be improved. As *T. asperellum* T-1 has been widely used to control plant diseases, it definitely possesses the ability to adapt to environmental changes. In addition, it was found to have a strong ability to degrade cellulose in our research. Therefore, the bio-control organism *T. asperellum* T-1 can be used not only as a practical decomposer of lignocellulose but also as an organic fertilizer producer by using lignocellulose as a raw material.

### The inhibiting effect of carbon source repression on cellulase production in *T. asperellum* T-1

We believed that glucose released from the wheat straw was accounting for the decline in enzymatic activity of both *T. asperellum* T-1 and *T.reesei* QM6a in Fig. 2, which may suppress the expression of the cellulose enzyme via substrate inhibition. This phenomenon is known as carbon source repression, and it ensures that the fungi do not waste too much gene expression and metabolic activity on the degradation of complex material when sugars that can be directly used are present. The production of cellulose- and hemicellulose-degrading enzymes has already been found to be controlled at the transcriptional level by the available carbon source in many lignocellulose-degrading strains [20]. In addition, carbon source repression may be the main reason for the poorer enzyme production yield and persistence of *T. asperellum* T-1. As previously illustrated, *T. asperellum* T-1 showed a better ability to degrade wheat straw than *T. reesei* QM6a at the early stage of fermentation, and *T. asperellum* T-1 might release more glucose than *T. reesei* QM6a during that period. Therefore, the carbon source repression caused by glucose appeared earlier in the fermentation of *T. asperellum* T-1 than that of *T. reesei* QM6a (Fig. 2). Then, a glucose inhibition experiment was further carried out to illustrate the carbon source repression in *T. asperellum* T-1. We added glucose at different concentrations into the fermentation system of *T. asperellum* T-1 at 0 h, 36 h and 60 h. These three time points were respectively the early stage of fermentation, the rising stage of cellulose production, and the stable period of cellulose production. The cellulase activity of the fermentation liquor was represented by the concentration of the reducing sugar released from filter paper and CMC-Na.

**Fig 3:**
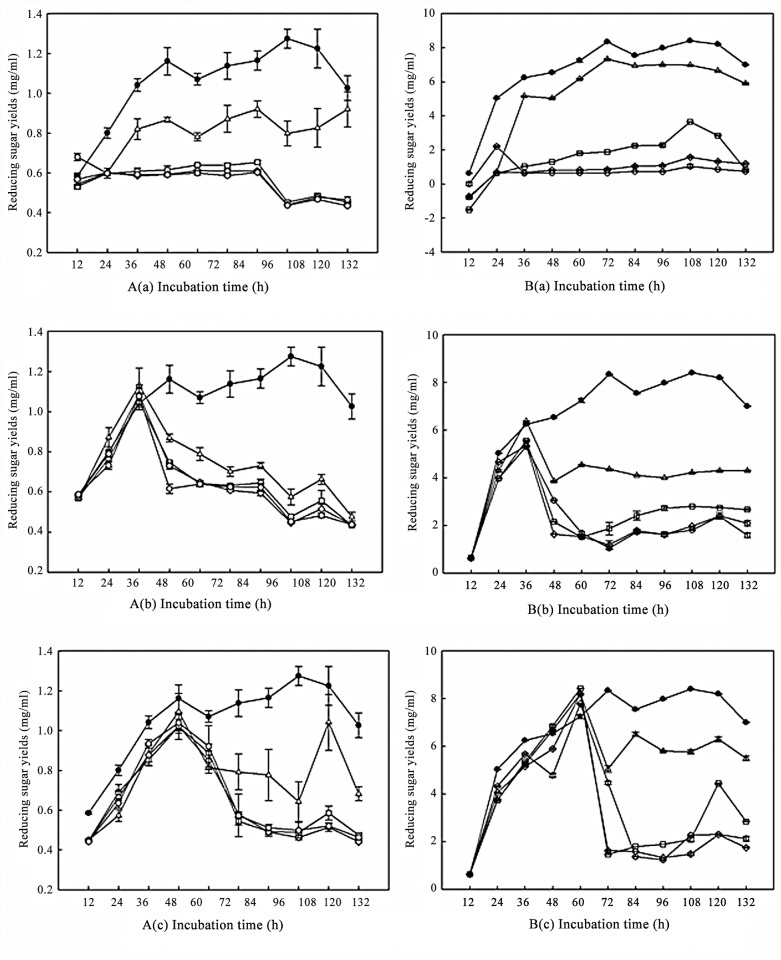
Enzyme production inhibition in *T. asperellum* T-1 by glucose addition at different growth periods, shown as the reducing sugar yield in the enzyme reaction system both in FPA (A) and CMCase (B). (A) FPA and (B) CMCase activity of *T. asperellum* T-1 with glucose added at different final concentrations (w/v) at 0 h (a), 36 h (b) and 60 h (c) of cultivation (0.5%: triangle up; 1%: square; 1.5%: diamond; 2.0%: hex; control: black circle). Error bars denote standard deviations from the mean values of triplicate measurements (n = 3).

Fig. 3 indicates that the inhibition effect of glucose at different concentrations was distinct. The 0.5% glucose had the smallest inhibition effect on enzyme production, while all of the glucose concentrations higher than 0.5% showed a stronger inhibitory effect on both FPase (Fig. 3A) and CMCase (Fig. 3B). This phenomenon occurred at all the three time points of 0 h, 36 h and 60 h. We found that the added glucose could all be exhausted by the mycelia within 48 h, except the group with 2% glucose added at 0 h of fermentation. It takes 60–72 h for that group to run all the glucose out (data not shown). After the glucose was exhausted, the cellulase production increased slightly. However, the glucose-inhibiting effect still lasted for a long time, as all the cellulase activities were significantly lower than that of the control group. In addition, the reducing sugar yields of FPase and CMCase in the groups with 0.5% glucose added had a comparatively more obvious increase than that in the groups with glucose concentrations higher than 0.5% (Fig. 3).

**Fig 4:**
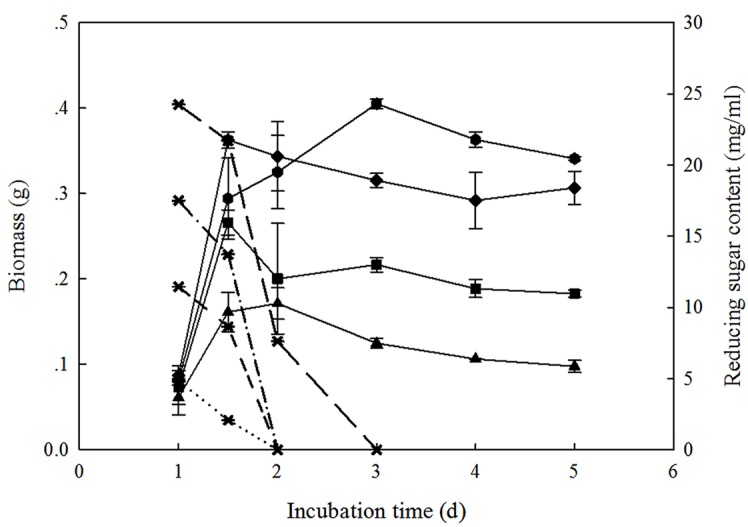
Biomass and glucose consumption when different concentrations of glucose were used as the carbon sources. (Biomass: 0.5%: black triangle up; 1.0%: black square; 1.5%: black diamond; 2.0%: black hex; glucose consumption: 0.5%: dotted; 1.0%: short-short; 1.5%: dash-dot; 2.0%: long dash). Error bars denote standard deviations from the mean values of triplicate measurements (n = 3).

In order to clarify the different repression effects of glucose at different concentrations in Fig. 3, the changing patterns of biomass and substrate consumption of strain T-1 were further investigated. Spores of *T. asperellum* T-1 (10^6^ spores ml^-1^) were cultured in media with glucose, at concentrations ranging from 0.5% to 2.0%, as carbon sources. According to Fig. 4, the cell growth increased as the carbon source concentration increased, showing biomass in the order of 2% glucose group>1.5% glucose group>1.0% glucose group>0.5% glucose group. The 2% glucose was exhausted within 3 days, while glucose lower than 2% only needed 2 days to be used up. When the glucose in the medium was exhausted, the cell growth ceased, and the biomass even decreased slightly. In view of the different inhibiting effects of glucose and the different enzyme activity recoveries after glucose was exhausted, as seen in Fig. 3, we thought there was no doubt that the addition of glucose could result in the decreasing expression of cellulase genes. However, the consistency of the carbon source repression and the poor recovery of enzyme activity were closely related to the glucose concentration and mycelia growth. Glucose of different concentrations used as carbon sources led to different mycelia growth. In particular, when compared with the groups with glucose higher than 0.5%, the fermentation systems with only 0.5% glucose added had a slower mycelia growth, which can be seen in Fig. 4. After the existing glucose was exhausted, the mycelia had much stricter growth requirements. Consequently, it was more urgent for the organism to secret cellulases to convert the wheat straw into glucose to satisfy their own mycelia growth requirements. In addition, we hypothesized that the substrate inhibition caused by the converted glucose further resulted in the consistent repression of cellulase production during the fermentation. The inhibition on cellulase biosynthesis caused by glucose is common in *Trichoderma* [21], and it has been studied comprehensively and thoroughly, especially in lignocellulose-degrading species such as *T. reesei*. However, research regarding carbon source repression in bio-control strains is still limited so far. We sought a deeper insight into carbon source repression in *T. asperellum* T-1 to lay a theoretical foundation for the strain’s improvement before it can be practically used in lignocellulose degradation.

### Repression of extracellular protein expression caused by glucose

**Fig 5:**
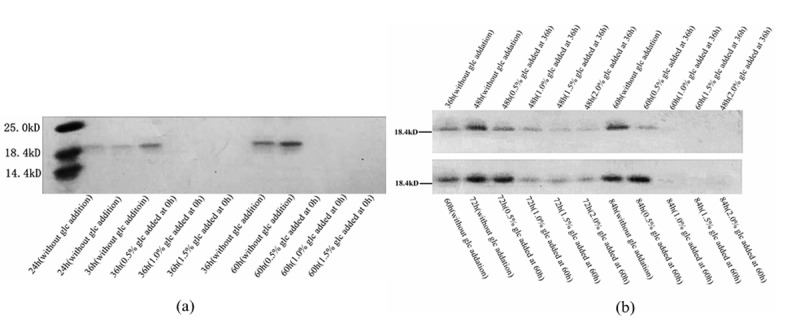
SDS-PAGE of fermented supernatant representing the protein expression differences when different concentrations of glucose were added at 0 h (a), 36 h and 60 h (b).

Carbon source repression can definitely inhibit the expression of extracellular secreted proteins in *T. asperellum* T-1, so SDS-PAGE was carried out to further illustrate it. Samples used in SDS-PAGE were taken from the fermentation systems of *T. asperellum* T-1 in the glucose repression experiment. As seen in Fig. 5, the added glucose clearly inhibited the expression of extracellular proteins. In the fermentation system without glucose addition, *T. asperellum* T-1 secreted a certain amount of extracellular protein, as the bands at approximately 18.4 kDa can been seen after cultivation for 24 h, 36 h and 60 h. And these bands became deeper with cultivation time increasing from 24 h to 60 h, indicating that the protein expression was increasing. However, when glucose was added, the band disappeared (Fig. 5a). This phenomenon also occurred when glucose was added at 36 h and 60 h of fermentation, but the band did not entirely disappear (Fig. 5b). From Fig. 5a, it seemed that the disappearance of protein bands had nothing to do with the concentration of glucose being added, as all of the groups had a band disappear after the addition of glucose. However, it is important to note that, in Fig. 5a, the samples were taken from the groups with glucose added at 0 h. This time point was of the initial fermentation stage, when there was less hyphae growth. While in Fig. 5b, after a cultivation for 36 h or 60 h, *T. asperellum* T-1 grew well and had secreted a certain amount of cellulases before glucose added. After 0.5% glucose was added, the concentration of glucose was so low that it could not show a strong enough inhibitory effect, so the band at approximately 18.4 kDa still could be seen clearly (Fig. 5b). However, if the added glucose concentrations were above 0.5%, this protein expression could be successfully suppressed, so that the band became shallow or disappeared (Fig. 5b). Therefore, we concluded that the existing glucose definitely has an inhibiting effect on the expression of cellulase gene, but the inhibiting intensity was closely related to the cell growth and the concentration of glucose.

Furthermore, the disappeared band in Fig. 5 was cut out and used in a preliminary secondary mass-spectrum identification (QSTAR Elite LC/MS/MS). The result showed that this protein shared an extensive homology with endo-1,4-β-xylanase I of *Trichoderma viride* (p<0.05, where p is the probability that the observed match is a random event; S1 Fig), and the coverage rate of the protein sequence was up to 49%. According to that result, we confirmed that the protein was endo-1,4-β-xylanase I, one type of cellulase cleaving the β-(1,4)-glycosidic bond between xylose units in the xylan backbone to produce xylo-oligosaccharides [22]. From the above, it is concluded that endo-1,4-β-xylanase I in *T. asperellum* T-1 was undoubtedly down-regulated in the process of carbon source repression. Actually, as early as 1989, endo-1,4-β-xylanase I was proved to be repressed by glucose in *T. reesei* [23]. In order to investigate more thoroughly, we decided to analyze the complete protein sequence of endo-1,4-β-xylanase I in *T. asperellum* T-1 by using bioinformatics methods. The coding sequence of the endo-1,4-β-xylanase I gene of *T. viride* was used as a probe to search for matching information in the protein database of *T. asperellum*. Then, an ORF of 786 bp (the protein sequence has been uploaded to NCBI; GenBank: KM277356) was found, including an intron sequence of 114 bp (S2 Fig). It encodes a 223-amino acid protein with a calculated *M*_*r*_ of 24.548 kDa. This discovery was in accord with the result in Fig. 5 that the band size was between 18.4 and 25.0 kDa. Furthermore, we believed that different carbon sources could induce the expression of different types of extracellular cellulases. In César Marcos Marcello’s research, when starch was used as a carbon source, *T. asperellum* expressed a high level of exo-1,3-β-glucanase, and the gene expression was also proven to be controlled by carbon source repression [11].

Quite unexpectedly, the coding sequence for endo-1,4-β-xylanase I in *T. asperellum* T-1 and in *T. viride* were of high homology, with only one different nucleic acid base at 16 bp. In *T. asperellum* T-1, this base was T (S2 Fig), while it was A in *T. viride*. This difference resulted in the functional proteins of these two filamentous fungi having only one correspondingly different amino acid. The corresponding amino acid was serine (Ser, S) in *T. asperellum* T-1, while it was threonine (Thr, T) in *T. viride*. However, we still did not figure out whether this minor difference could result in structural and functional changes of the protein. In fact, *T. asperellum*, which produces finely warted conidia, was initially described as a new species in 1999. Then, it was differentiated from *T. viride* because *T. viride* produces conspicuously warted conidia. However, S. Sriram’s research found that *T. viride* is actually *T. asperellum* or its cryptic species *T. asperelloides* [24], which might explain the extremely high similarity of those two genes in our research. On the other hand, this discovery may further provide an evidence for the conjecture that *T. asperellum* and *T. viride* may be the same species.

### Characteristics of the *cre* gene and amino acid sequence comparison

It is known that carbon source repression in microorganisms is a way to control the synthesis of a range of enzymes required for the utilization of less-favored carbon sources when more readily utilized carbon sources are present in the medium [25]. Functional factors such as Cre and Ace [20], which play key roles in this process, have been investigated in many species. The functional factor Cre undoubtedly plays the most important regulatory role, and its protein sequences in many different species always share a highly homologous region with DNA-binding ability. The Cre proteins can bind onto the promoter of related genes to obstruct the gene expression. A protein called CreA in *A. niger* was proven to participate in the catabolite repression of the transcription of genes involved in proline use, ethanol metabolism and polysaccharide hydrolysis [26], and its homologous protein, called Cre1 in *T. reesei*, also plays the main regulatory role in carbon source metabolic repression [27]. Here, we called the homologous protein ‘Cre1’ in *T. asperellum* T-1 because of the relatively close relationship between *T. asperellum* and *T. reesei*.

As carbon source metabolic repression is widespread in filamentous fungi and the known *cre* genes in different species always have high homologies, we obtained the *cre1* gene sequence using bioinformatics methods. The sequence of the *cre1* gene in *T. reesei* was used as the probe to search for target homologous nucleic acid information in the genomic sequence of *T. asperellum*. An ORF encoding a protein of 402 amino acid residues with a predicted molecular mass of 44.238 kDa was isolated. The sequence validity was identified through PCR and DNA sequencing, and the obtained sequence has been uploaded to NCBI (GenBank: KM277357). The protein sequence of Cre1 showed a high similarity to the sequences of Cre proteins from *T. reesei* and *A. niger*, with similarities of 86% and 54%, respectively. Multiple protein sequence alignments of the Cre protein among *T. asperellum* T-1, *T. reesei*, *A. niger* and *S. cerevisiae* are given in S3 Fig. to compare their structures. The amino acid sequence AA_57_-AA_114_ (indicated by a rectangular flag in S3 Fig) of Cre1 in *T. asperellum* T-1 was the typical C_2_H_2_-type Zn-fingers, which plays the binding role during the regulation of the corresponding cellulose-coding genes. As seen in S3 Fig, these four sequences were of high homology. AA_263_-AA_306_ (underlined in S3 Fig) of *T. asperellum* T-1 was a stretch that showed 100% and 97.7% identity, respectively, to *T. reesei* and *A. niger*, and these regions showed significant similarity with the *S. cerevisiae* RGR1 [28]. Nucleic acid sequence analysis of the *cre1* gene in *T. asperellum* T-1 indicated that this gene has no putative intron (data not shown), but the Ala-rich region (indicated by an oval tag in S3 Fig) observed immediately after the Zn-fingers region in *A*. *nige*r CreA was absent in *T. asperellum* T-1. All of the structural features above were closely involved normal regulatory functions. In fact, studies about carbon source repression in fungi have been conducted for a long time, and the phenomenon was recently found to be more complex. More regulatory factors were proven to be involved in this process, and the existing regulatory mechanism functioning by known regulatory factors has been constantly supplemented or corrected. For example, Roy et al. found that CreA in *Aspergillus nidulans* must require some modification or interaction to act as a repressor instead of requiring transcriptional auto-regulation, regulated intracellular localization, or degradation of CreA [29]. However, almost all these studies are focusing on strains having lignocellulose degrading ability, with a purpose of improving their degradation ability. Here, we shifted our attention to the bio-control strain for their practicality, and we aim to provide theoretical references for the improvement of T-1 to be used in lignocellulose degradation.

### Prediction of Cre1 binding sites and features of the endo-1,4-β-xylanase I gene promoter

As mentioned above, Cre proteins conduct their regulation function through recognizing a certain DNA sequence, and then they can bind onto the promoters of target genes. Previous studies concerning the repressor Cre1/CreA from various fungi [25, 26, 30, 31] showed that the consensus sequence for Cre protein binding is considered to be 5’-SYGGRG-3’ (S = C or G, Y = C or T, R = A or G). As our research has proven, the expression of endo-1,4-β-xylanase I gene is controlled by Cre1 in *T. asperellum* T-1 under carbon source repression. Then, in order to elucidate the interaction between Cre1 and the endo-1,4-β-xylanase gene, the promoter sequence approximately 1 kb upstream of the endo-1,4-β-xylanase I ORF was analyzed (S4 Fig). As shown in S4 Fig, five sets of sequences conforming to the consensus sequence of 5’-SYGGRG-3’ were found, including 5’-CTGGAG-3’, 5’-GCGGGG-3’, 5’-GTGGCG-3’, 5’-CCGGGG-3’ and 5’-CTGGCG-3’ at positions-170 bp, -198 bp, -300 bp, -332 bp and-628 bp, respectively. However, it is important to note that not all the sequences conforming to the consensus sequence can function as the binding site. The consensus sequence can be found in various types of cellulose enzyme gene promoter sequences [26]; for example, the binding site of Cre1 on cellobiohydrolase I (cbhl) in *T. reesei* is 5′-GCGGAG-3′ [32].

Moreover, observation of the sequence depicted in S4 Fig revealed the presence of two TATA-like sequences at-551 bp and-908 bp from the major starting point of transcription (ATG of ORF). Additionally, two sets of CAAT-boxes were found at-282 bp and-899 bp next to one of the TATA-like sequences. It is well known that both types of structures are the core sequence of the promoter for the start of gene expression. In addition, the 5’ region of the endo-1,4-β-xylanase I gene contains large blocks of (C+T)-rich sequences from-438 bp to-405 bp, which was reported common around the start points of transcription of many fungal genes [33]. We believe that the structural and functional analysis of the endo-1,4-β-xylanase I gene promoter can make its regulation better understood.

Even though the transcriptional repressors are always of high homology, there must be a number of different factors and mechanisms involved in regulation among different filamentous fungi. As previously noted, in eukaryotic organisms, appropriate transcriptional regulation often requires the combinatorial and synergistic action of different repressors and activators, and Cre1 is only one of the most common repressors.

## Conclusion

*T. asperellum* T-1 is a bio-control strain, while in our study, it was initially demonstrated to be a potential decomposer of lignocellulose. Its characteristics of robust growth, strong spore production, rapid enzyme production and good pH tolerance allow T-1 to effectively avoid microbial contamination during industrial fermentation and make T-1 more suitable for practical applications. In addition, a bio-control strain with a strong ability to degrade lignocellulose can be used as an organic fertilizer producer or as a healthy soil fungus, which can convert lignocellulose into humus and also control plant diseases in soil. The Cre1-mediated carbon source repression caused by glucose was firstly illustrated in the bio-control *T. asperellum* T-1, and the repression effect resulted in the expression of endo-1,4-β-xylanase I decreased obviously when glucose exists. Owing to the similarity of regulation mechanism on cellulase expression between *T. asperellum* T-1 and the known *T. reesei*, our works suggested that the current engineering methods and techniques developed for *T. reesei* might provide good references for improvement of *T. asperellum T-1* being used in lignocellulose degradation.

## Supporting Information

S1 Fig Identification results of the secondary mass spectrum of the band in Fig. 5. The protein score exceeds 400 in our result, where scores greater than 59 indicate identity or extensive homology.
(TIF)

S2 Fig Gene sequence of endo-1,4-β-xylanase I in *T. asperellum* T-1. Intron sequences: double underline; different nucleotide of endo-1,4-β-xylanase I compared with that in *T. viride*: blue rectangle.
(PDF)

S3 Fig Multiple protein sequence alignment of Cre1 in *T. asperellum* T-1 with Cre1 in *T. reesei*, CreA in *A. niger* and Mig1p in *S. cerevisiae* including the Zn-fingers domain (rectangular flag); Ala-rich region in CreA of *A. niger* (oval tag) and the stretch structure (underline). (TIF)

S4 Fig 1 kb upstream nucleic acid sequence analysis of the endo-1,4-β-xylanase I gene. (TATA-box: double underline; CAAT-box: double tilde; candidate recognition sequences of Cre1: rectangle; start point of ORF: rounded rectangle).
(TIF)
