# Correction: Characterization of Cellulase Secretion and Cre1-Mediated Carbon Source Repression in the Potential Lignocellulose-Degrading Strain *Trichoderma asperellum* T-1

**DOI:** 10.1371/journal.pone.0127492

**Published:** 2015-04-29

**Authors:** The PLOS ONE Staff

The Funding section was unintentionally omitted. The complete, correct funding information is:

This study was supported by the National Natural Science Foundation of China (41271335; 31470191), the Major State Basic Research Development Program of China (973 program) (2015CB150502), the High Technology Research and Development Program of China (863 Program) (2012AA06A203), and the National Key Technology Rand D Program (2012BAC17B04), the Science and Technology Project of Zhejiang Province (2011C13016; 2013C3303; 2014C33019), and the Environmental Science Project of Zhejiang Province (2012B006). The funders had no role in study design, data collection and analysis, decision to publish, or preparation of the manuscript.

The publisher apologizes for the error.
