# Supplementary material for: Characterization of Cellulase Secretion and Cre1-Mediated Carbon Source Repression in the Potential Lignocellulose-Degrading Strain Trichoderma asperellum T-1
Source: PLoS One. 2015 Mar 5;10(3):e0119237. doi: 10.1371/journal.pone.0119237 (PMC4351060; doi:10.1371/journal.pone.0119237)
Supplement: S2 Fig — Intron sequences: double underline; different nucleotide of endo-1,4-β-xylanase I compared with that in T. viride: blue rectangle. (PDF) [file pone.0119237.s002.pdf]

ATGGTCTCCTTCACATCTCTCCTCGCCGGCTTCGTCGCTGTTACCGGAGTT

CTGTCCGCTCCCACTGAGAACGTGGAGGTCGTGGACGTGGAGAAGCGCCA

GACGATTGGCCCCGGCACTGGCTTCAACAACGGCTACTACTACTCGTACT

GGAACGATGGCCATTCCGGCGTGACATACACCAACGGTGCTGGCGGCTCA

TTCAGCGTCAACTGGGCAAACTCGGGCAACTTTGTCGGAGGCAAGGGATG

GAACCCCGGCAGCAGCTCCAGGTAAGTGACTCAAGTGAGGAACACAACT

CAGATACTCCAAGACACTGGCCACCAGTACTCCCCTGTGCCATATTCACGT

GCAGAGAGCTAATACCACTCGCTCTGTTAATCAAGGGTCATCAACTTCTCT

GGCAGCTACAACCCCAACGGCAATAGCTACCTCTCAGTCTATGGCTGGTC

AAAGAACCCTCTCATCGAGTACTACATCGTTGAGAACTTTGGAACCTACA

ACCCATCGACCGGCACCACCAAGCTGGGCGAGGTGACCTCTGACGGCAGC

GTCTACGACATCTACCGCACGCAGCGAGTCAACCAGCCTTCCATCATCGG

AACCGCCACCTTTTACCAGTACTGGTCTGTCCGCCGCAACCACCGCTCCAG

CGGCTCGGTCACGGTTGCGAACCACTTCAACGCGTGGCGCAACCTGGGCT

TGACCCTGGGAACCTTGGACTACCAGATCATTGCCGTGGAGGGCTACTTT

AGCTCTGGTAACGCCAACATTAACGTTAGCTAA
