# Supplementary figures and images for: Characterization of Cellulase Secretion and Cre1-Mediated Carbon Source Repression in the Potential Lignocellulose-Degrading Strain Trichoderma asperellum T-1
Source: PLoS One. 2015 Mar 5;10(3):e0119237. doi: 10.1371/journal.pone.0119237 (PMC4351060; doi:10.1371/journal.pone.0119237)

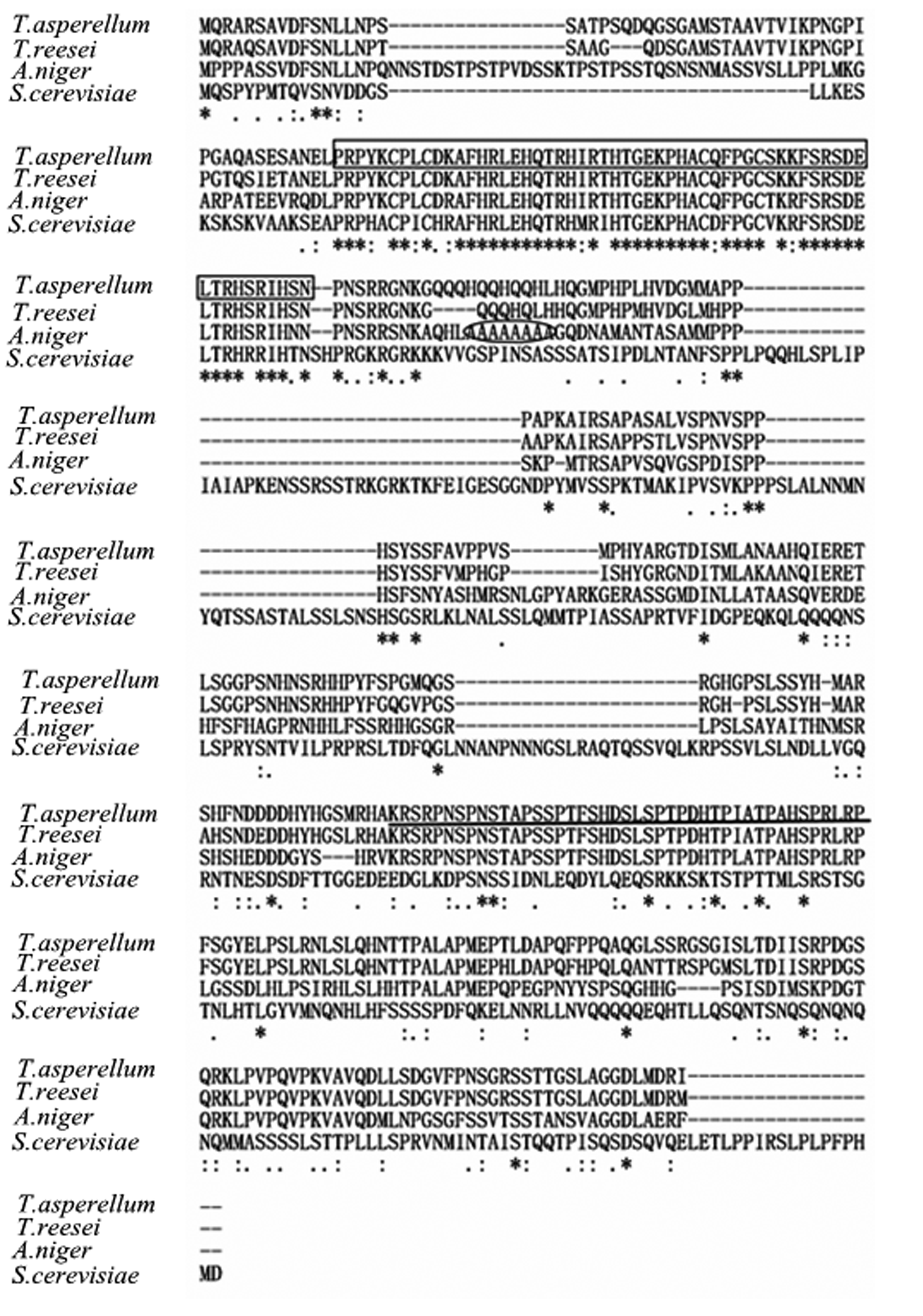

Supplement: S3 Fig — (TIF) [file pone.0119237.s003.tif]

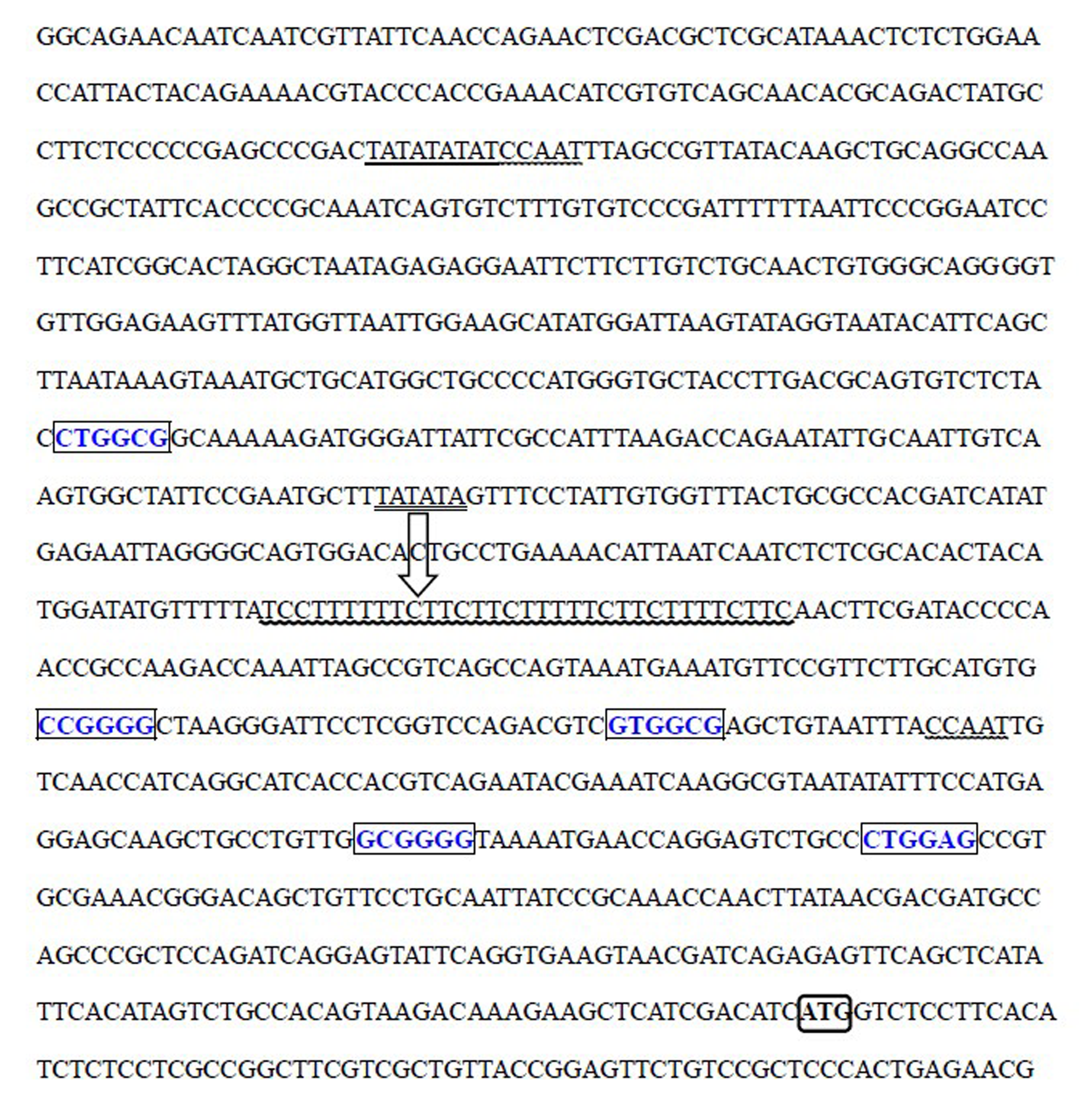

Supplement: S4 Fig — (TATA-box: double underline; CAAT-box: double tilde; candidate recognition sequences of Cre1: rectangle; start point of ORF: rounded rectangle). (TIF) [file pone.0119237.s004.tif]
